# *ComBat-seq*: batch effect adjustment for RNA-seq count data

**DOI:** 10.1093/nargab/lqaa078

**Published:** 2020-09-21

**Authors:** Yuqing Zhang, Giovanni Parmigiani, W Evan Johnson

**Affiliations:** Department of Bioinformatics and Clinical Data Science, Gilead Sciences, Inc., 333 Lakeside Dr, Foster City, CA 94404, USA; Department of Data Sciences, Dana-Farber Cancer Institute, 450 Brookline Ave, Boston, MA 02215, USA; Department of Biostatistics, Harvard T.H. Chan School of Public Health, 677 Huntington Ave, Boston, MA 02115, USA; Division of Computational Biomedicine, Boston University School of Medicine, 72 East Concord Street, Boston, MA 02118, USA; Graduate Program in Bioinformatics, Boston University, 24 Cummington Mall, Boston, MA 02215, USA; Department of Biostatistics, Boston University School of Public Health, 715 Albany Street, Boston, MA 02118, USA

## Abstract

The benefit of integrating batches of genomic data to increase statistical power is often hindered by batch effects, or unwanted variation in data caused by differences in technical factors across batches. It is therefore critical to effectively address batch effects in genomic data to overcome these challenges. Many existing methods for batch effects adjustment assume the data follow a continuous, bell-shaped Gaussian distribution. However in RNA-seq studies the data are typically skewed, over-dispersed counts, so this assumption is not appropriate and may lead to erroneous results. Negative binomial regression models have been used previously to better capture the properties of counts. We developed a batch correction method, ComBat-seq, using a negative binomial regression model that retains the integer nature of count data in RNA-seq studies, making the batch adjusted data compatible with common differential expression software packages that require integer counts. We show in realistic simulations that the ComBat-seq adjusted data results in better statistical power and control of false positives in differential expression compared to data adjusted by the other available methods. We further demonstrated in a real data example that ComBat-seq successfully removes batch effects and recovers the biological signal in the data.

## INTRODUCTION

Genomic data are often produced in batches due to logistical or practical restrictions, but technical variation and differences across batches, often called batch effects, can cause significant heterogeneity across batches of data (1). Batch effects often result in discrepancies in the statistical distributions across data from different technical processing batches, and can have unfavorable impact on downstream biological analysis. The presence of batch effects often reduces the benefits of integrating batches of data to increase the inferential power to discover relevant biology from the combined data.

Batch effects often cannot be fully addressed by normalization methods and procedures. The differences in the overall expression distribution of each sample across batch may be corrected by normalization methods, such as transforming the raw counts to (logarithms of) CPM, TPM or RPKM/FPKM, the trimmed mean of M values (TMM) (2), or relative log expression (RLE) (3). However, batch effects in composition, i.e. the level of expression of genes scaled by the total expression (coverage) in each sample, cannot be fully corrected with normalization. An example of composition batch effects was provided in microarray data (1), showing that while the overall distribution of samples may be normalized to the same level across batches, individual genes may still be affected by batch-level bias.

Many methods have been proposed to address batch effects in RNA-seq studies. For example, ComBat (4) remains one of the most popular batch effect adjustment methods when the effects come from known sources. For heterogeneity from unknown sources, SVASeq (5) and RUVSeq (3) are commonly used. Methods designed for specific downstream tasks have also been proposed, including our own work using reference batches for biomarker development and training (6). For differential expression, many common methods or procedures (e.g. edgeR (7) and DESeq2 (8)) suggest to include batch variables as covariates in the linear models behind these methods to account for the impact of batch.

Despite the established progress, there are still gaps in batch adjustment methodology for RNA-seq data which need to be bridged. Often times, batch effect adjustment methods do not adequately model the complexity of batch-to-batch heterogeneity, do not directly provide adjusted data with batch effects removed, or do not preserve the integer nature of counts in the adjusted data, despite the requirement of software such as edgeR and DESeq2 that specifically require untransformed count matrices as inputs. This results in an inconsistency in the analysis pipeline of RNA-seq studies, as batch corrected data cannot be used as inputs for these differential expression software. For these practical issues, it is favorable to develop a method which generates adjusted data and is able to preserve the count nature of data.

More importantly, many popular adjustment methods, including ComBat, assume Gaussian distributions for the underlying distribution of the data, which is not an appropriate distributional assumption for counts. These methods typically estimate parameters representing differences in the statistical moments across batches (usually the mean and the variance). Then they adjust all batches to the same overall level in these moments. Such adjustment does not preserve integers, and may results in negative values in adjusted count matrix, which is difficult to interpret biologically. In addition, it has been well-established that there exists a mean-variance dependence in RNA-seq count data (9). Distributions of counts are skewed and over-dispersed, i.e. the variance is often larger than the mean of gene expression and genes with smaller counts tend to have larger variances. These properties cannot be reflected with Gaussian distribution, which assumes independent mean and variance parameters. Negative binomial regression models have been widely used to model count data in RNA-seq studies. The Negative binomial distribution has the potential to describe the skewness and mean-variance relationship observed in count matrices. We propose to extend the original ComBat framework to RNA-seq studies using negative binomial regression.

Finally, existing methods may not be flexible enough to address all types of batch effects. In particular, including batch variables in software for differential expression may be sufficient to account for batch effects in the mean expression. However, since both software assume a single dispersion parameter for all samples, variance batch effects is restricted, and completely determined by mean batch effects, due to the properties of negative binomial modeling. Such assumption is strong and may not always hold for real data. Therefore, we propose a more flexible approach to address batch effects in the variance.

In this paper, we present a batch effect adjustment method, ComBat-seq, that extends the original ComBat adjustment framework to address the challenges in batch correction in RNA-seq count data. It generates adjusted data in the form of counts, thus preserving the integer nature of data. We demonstrate that ComBat-seq adjustment has potential benefits in differential expression compared to the other adjustment methods, especially when there is a large variance batch effect in the data.

## MATERIALS AND METHODS

**Figure 1.**
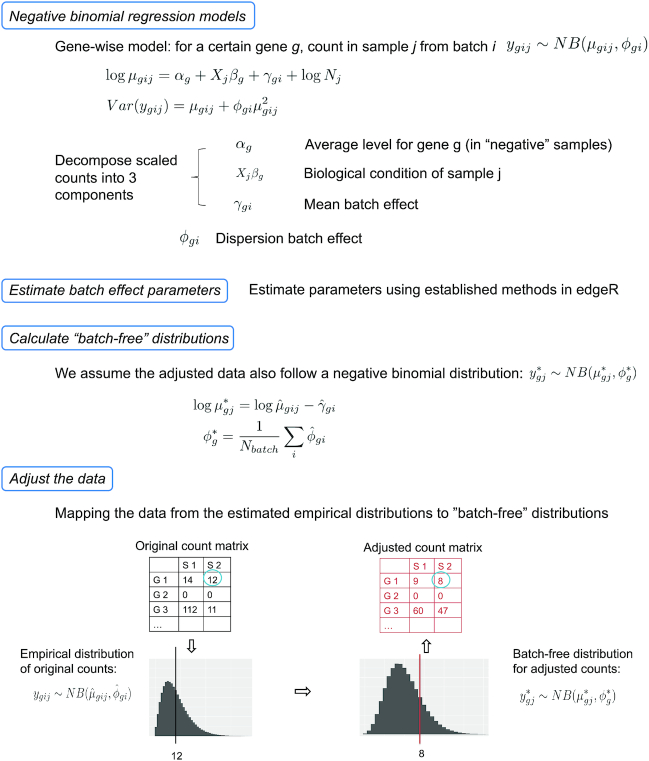
A diagram for the ComBat-seq modeling and adjustment workflow.

We propose ComBat-seq, which uses a negative binomial regression model to estimate batch effects based on the count matrix in RNA-seq studies. Parameters of the regression model are estimated using established methods (7,10–11). With the estimated batch effect parameters, we calculate ‘batch-free’ distributions, i.e. the expected distributions if there were no batch effects in the data based on the model. We then adjust the data by mapping the quantiles of the empirical distributions of data to the batch-free distributions. Since we assume batch-free distributions to also be negative binomial, the adjusted data remain integer-valued and can be used as inputs for popular differential expression software. See sections below and Figure 1 for details of ComBat-seq. Finally, one of the advantageous features of ComBat is the hierarchical empirical Bayes modeling, which pools information across genes for parameter estimation, making the adjustment robust for data with small sample sizes and/or outlying values (4). In the ComBat-seq software, we provide a similar option to share information across genes. We also evaluated the method, with details summarized in Supplementary Information and Supplementary Figure S1.

### ComBat-seq model

We define a regression model for each gene. Let the expression count value for gene *g* of sample *j* from batch *i* be denoted by *y*_*gij*_. We assume that *y*_*gij*_ follows a negative binomial distribution *NB*(μ_*gij*_, ϕ_*gi*_), where μ_*gij*_ and ϕ_*gi*_ are the mean and the dispersion parameters. We propose the gene-wise model:

$$\begin{eqnarray*} \log \mu _{gij} &=& \alpha _g + X_j\beta _g + \gamma _{gi} + \log N_j \\ var(y_{gij}) &=& \mu _{gij} + \phi _{gi}\mu _{gij}^2 \end{eqnarray*}$$

where α_*g*_ denotes the logarithm of expected counts for ‘negative’ samples. *X*_*j*_β_*g*_ reflects changes to the log of expected counts due to biological conditions, which is preserved in the data after adjustment. In this term, *X*_*j*_ may be an indicator of the biological condition for sample *j*, or a continuous value for a clinical covariate. β_*g*_ denotes the corresponding regression coefficient. *N*_*j*_ represents the library size, i.e. total counts across all genes in sample *j*. The mean and dispersion batch effect parameters are denoted by γ_*gi*_ and ϕ_*gi*_, respectively, modeling the effect of batch *i* on gene *g*. We estimate the model parameters, especially batch effect parameters γ_*gi*_ and ϕ_*gi*_, following the established methods in edgeR (7,10–11). Specifically, the mean batch effect parameters γ_*gi*_s are estimated with Fisher scoring iteration, implemented in an optimized way to reduce the computational time. The dispersion parameters ϕ_*gi*_ are estimated gene-wise by maximizing the Cox–Reid adjusted profile likelihood (APL) (11), and results in non-negative dispersion estimates. Note that the estimates for mean of expression are not required to be non-negative, since they are on the log scale. We estimated the gene-wise dispersion within each batch in ComBat-seq.

### ComBat-seq adjustment

After modeling, we obtain estimated batch effect parameters $\hat{\gamma }_{gi}$ and $\hat{\phi }_{gi}$, as well as the fitted expectation of the count $\hat{\mu }_{gij}$. We then calculate parameters for ‘batch-free’ distributions as follows: we assume that the adjusted data $y_{gj}^*$ follows a ‘batch-free’ negative binomial distribution $NB(\mu _{gj}^*, \phi _{g}^*)$, where parameters are calculated as

$$\begin{eqnarray*} \log \mu _{gj}^{*} &=& \log \hat{\mu }_{gij} - \hat{\gamma }_{gi}\\ \phi _g^* &=& \frac{1}{N_{batch}} \sum \limits _i \hat{\phi }_{gi} \end{eqnarray*}$$

Then, the adjusted data $y_{gj}^*$ is calculated by finding the closest quantile on the batch-free distribution to the quantile of the original data *y*_*gij*_ on the empirical distribution, estimated as $NB(\hat{\mu }_{gij}, \hat{\phi }_{gi})$. Specifically, we find the adjusted value $y_{gj}^*$ such that $F^*(y_{gj}^*) = P(y^* \le y_{gj}^*)$ is closest in absolute value to *F*(*y*_*gij*_) = *P*(*y* ≤ *y*_*gij*_). Zero counts are mapped to zeros. We perform this mapping for every value in the count matrix, which completes the adjustment.

### Simulations

We evaluated the performance of ComBat-seq with simulation experiments consisting of three steps: (i) we simulated RNA-seq studies with biological conditions and batch effects, (ii) adjusted the batch differences with ComBat-seq as well as other available methods and (iii) evaluated the performance of batch effect adjustment by the impact on differential expression using the adjusted data.

We used the *polyester* R package (12) to simulate realistic RNA-seq studies, which are in the form of gene-by-sample count matrices. We designed two biological conditions and two batches of samples. The *polyester* package human genome reference example provides information for 918 genes which we divided them into two groups: group 1 has higher expression in batch 2 and lower in batch 1, while group 2 has the reversed pattern, higher in batch 1 and lower in batch 2. This forms a batch effect in the ‘composition’ of expression as described in the Introduction section, which cannot be fully addressed by normalization. We assume a biological variable with two levels, ‘negative (0)’ and ‘positive (1)’, which can represent ‘control’ and ‘tumor’ samples in a real dataset, for example. We simulated both upregulated and down-regulated true differentially expressed (DE) genes in both gene groups with increased expression in the positive (upregulated) or the negative (downregulated) biological condition. The remaining genes are only affected by batch, not by the condition. Differences in the average count of genes are simulated by specifying fold changes across biological and batch sample groups using *polyester*. We also made the dispersion of batch 2 a number of times larger than that of batch 1, allowing for different true dispersion parameters across batches. Supplementary Figure S2 shows the design we used for simulated data.

We repeated the simulation while varying the level of mean and dispersion batch differences. Specifically, we changed the parameters for simulation such that mean of batch 2 is 1.5, 2 or 3 times that of batch 1. The dispersion of batch 2 was set to be 2-, 3- or 4-fold of that of batch 1. Experiments with no mean batch effect or no dispersion differences were also included. Results were averaged over 300 repeated simulations under each parameter setting.

Our selected parameters in simulations are consistent with the degree of batch effect in real data, as summarized in Supplementary Table S1. Our observed condition signal (fold change in expression) from real studies range from 1.65 to 3.98, and we specified a biological signal of 2-fold in simulations. With regard to batch differences in the moments, we observed mean batch effect to be in the range of 1.62- and 1.88-fold, and variance difference to be in 1.26- to 7.09-fold. Our selected parameters in the simulations align with the realistic range, suggesting that the results are likely representative for the expected effect on real data batch adjustment.

The batch effects in mean and dispersion (variance) were adjusted with ComBat-seq, the ‘one-step’ approach, i.e. to include the batch variable in differential expression linear models, as well as with SVA-seq and RUV-seq. We also included another commonly used method in practice, which is to transform the count matrix to logCPM, then use the batch correction methods designed for Gaussian distributed data, such as the original ComBat method. For SVA-seq, we computed a single surrogate variable, then included it as a covariate in downstream differential expression. For RUV-seq, we used the RUVg method, and randomly sampled 10 genes that we simulated to not respond to the biological condition, and used them as negative control genes. We selected only one surrogate/latent variable, to reflect the design of the simulated study, where we simulated only biological and batch differences.

Aside from batch adjusted data, we included two additional experiments for comparison: differential expression performed on (i) data without simulated batch effects, and (ii) data with simulated batch differences, but no adjustment. We compared both the statistical power (true positive rate, TPR) and control of type-I errors (false positive rate, FPR) in detection using data without batch effects, data with batch effects before and after different adjustments.

### Real data application

We applied the proposed ComBat-seq approach on an RNA-seq data from a perturbation experiment using primary breast tissue attempting to profile the activity levels of growth factor receptor network (GFRN) pathways in relation to breast cancer progression (13,14). We took a subset of experiments, which consists of three batches. In each batch, the expression of a specific GFRN oncogene was induced by transfection to activate the downstream pathway signals (different oncogene/pathway in each batch). Controls were transfected with a vector that expresses a green fluorescent protein (GFP), and GFP controls were present in all batches. More specifically, batch 1 contains five replicates of cells overexpressing HER2, and 12 replicates for GFP controls (GEO accession GSE83083); batch 2 contains six replicates of each for EGFR and its corresponding controls (GEO accession GSE59765); batch 3 consists of nine replicates of each for wild-type KRAS and GFP controls (GEO accession GSE83083).

Note that this is a challenging study design for batch effect adjustment: the control samples are balanced across batches, while each of the 3 kinds of treated cells, with different levels of biological signals, is completely nested within a single batch. A favorable adjustment would pool control samples from the three batches, while keeping all treated cells separated from the controls and from each other.

We combined the three batches and performed batch correction. Among the batch correction methods considered, only RUV-seq, the original ComBat used on logged and normalized data and ComBat-seq output adjusted data. We apply these methods to address the batch effects in the pathway signature dataset. We compared ComBat-seq with the other methods, both qualitatively through principal component analysis (PCA) and quantitatively with explained variations by condition and batch.

The ‘one-step’ approach and SVA-seq are not considered in PCA because they do not generate adjusted data after batch correction. For RUV-seq, we do not know which genes are appropriate for negative control genes, unlike in the simulation studies. Therefore, we used the RUVs method, which is more robust to the choices of negative control genes than RUVg (3). We computed the least DE genes within each batch for the 3 activated pathways (FDR > 0.95), and took the overlapping genes across pathways as the negative controls.

## RESULTS

In the sections below, we justify the necessity of using negative binomial distribution instead of Gaussian distribution for count data. We develop and implement ComBat-seq, and apply the method to the simulated and real data examples. We then summarize our observations, showing the potential benefits of ComBat-seq adjustment.

### Using appropriate model assumptions for count data

**Figure 2.**
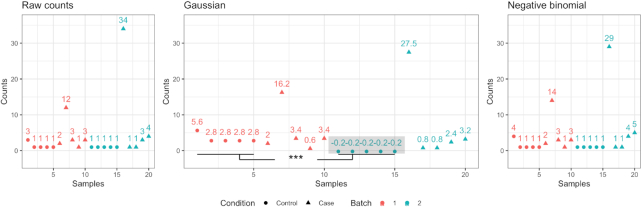
Problematic results caused by applying a Gaussian-based batch adjustment on count data. We simulated a count matrix with a balanced case-control design and two batches. The first panel shows the counts for a simulated gene which is expressed at low levels in most cases and control samples. However, one case sample in each batch, especially in the second batch, contains a large value. Adjustment based on a Gaussian distribution brings the mean of the two batches to the same level, causing artificially induced differences across control samples from the two batches (*P*-value = 0.0033). When applying ComBat-seq based on negative binomial distribution, the adjusted data no longer contain the negative values (shown in gray box) or the erroneous significant difference between control samples from the two batches.

An example demonstrating the weakness of Gaussian-based models is given in Figure 2. In this example, we simulated a count matrix using *polyester* (12) with balanced case-control design and two batches. Figure 2 shows the raw and batch adjusted counts for a single gene. The control samples in both batches have low expression, while there is a case sample in batch 2 with a relatively large count (over 30). When estimating the differences in mean across batch, due to the sample with the large count in the second batch, the mean of the second batch is estimated to be larger than that of batch 1. If we apply Gaussian-based batch adjustment which brings the mean to the same level, control samples in the second batch will be adjusted to negative values, while counts in the first batch will be increased. This results in a significant artificial difference between control samples from the two batch after correction (*P* = 0.0033). These observations demonstrate the potential issue of applying batch correction method using Gaussian distribution on count data. A more appropriate model for integer counts would avoid such limitations.

We then applied the ComBat-seq method, which assumes negative binomial distributions for the underlying data. As shown in Figure 2, the adjusted data do not contain negative values or the false significant result between the control samples of the two batches. This suggests that the negative binomial assumption is robust to outliers such as these and indeed addresses the limitations mentioned above.

### Simulations

**Figure 3.**
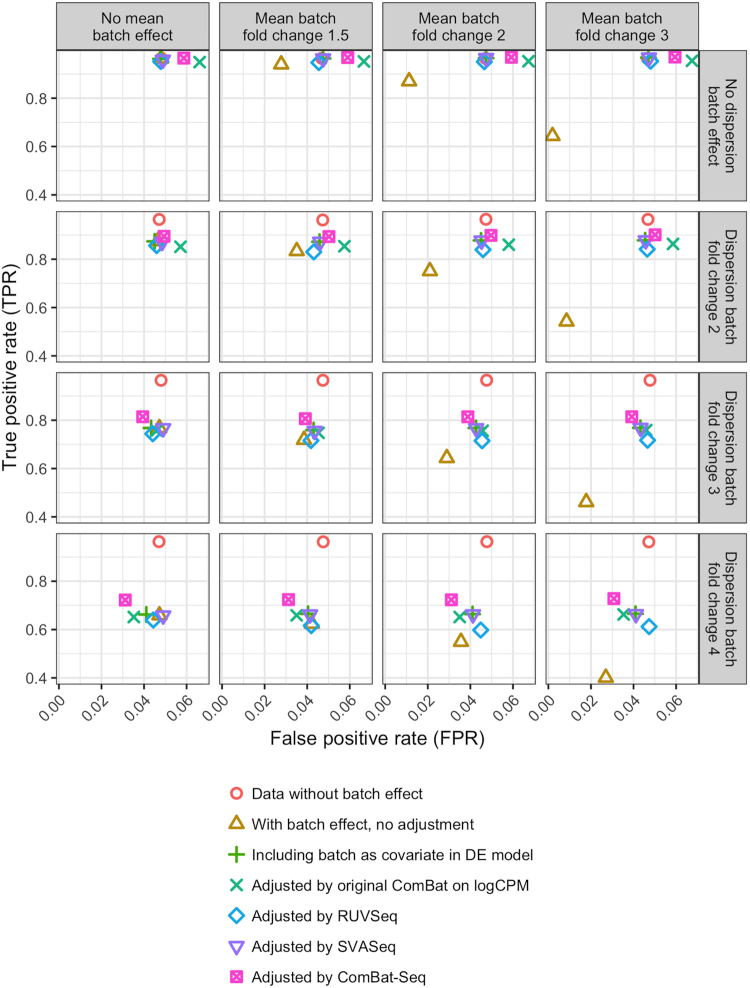
Simulation results under increasing level of differences across batch in the mean and variance of expression. Batch effects in the mean or the variance will cause a loss of power for differential expression detection. While all methods are able to increase the power for analysis, ComBat-seq generally achieves the best power. Also, when there is a sufficient level of dispersion differences across batch, ComBat-seq is able to better control false positives than the other methods.

We evaluated ComBat-seq and compared its performance with other available approaches in our simulation studies as described in the Method section. Results comparing all batch adjustment methods under different settings of degree of batch effects are summarized in Figure 3.

Having batch effects in the data causes decrease in both true and false positive rates, compared to data without batch effects (FPR: 0.048, TPR: 0.96). For example, having only batch effects in the mean of at 1.5-fold but no dispersion differences reduces FPR to 0.028, and TPR to 0.94. Having a 2-fold dispersion batch effect but no mean batch effect results in a 0.046 FPR and a 0.88 TPR.

ComBat-seq is able to control false positive rates under 0.05 when there are dispersion differences, but for the unrealistic case where there is no dispersion difference across batches both ComBat-seq and ComBat on logCPM have FPRs in the 0.059–0.067 range. Other methods are able to control the FPR under 0.05 on all cases. Therefore existing methods, such as including batch as a covariate in the differential expression methods, may be sufficient to address batch effects in the mean. In this case, ComBat-seq which assumes separate dispersion across batch may be redundant and lead to higher false positives. In all other scenarios, which are more common in real data, ComBat-seq is able to control false positive rates. When there are large dispersion differences across batch, which often occurs when combining heterogeneous batches, e.g. from different studies or profiling platforms, ComBat-seq is more conservative than the other methods, though almost all methods have an appropriate level of false positive control. For example, when applied on data with no mean batch effect and a 3-fold dispersion differences, ComBat-seq generates the the most conservative FPR of 0.039, compared to the other methods (including batch as a covariate: 0.043, original ComBat on logCPM: 0.046, RUV-seq: 0.044, SVA-seq: 0.049). The false positive rates using data adjusted by ComBat-seq further decrease as the level of dispersion difference increases (0.031 at the 4-fold dispersion difference, compared to the 0.039 FPR at the 3-fold difference).

In a realistic range of a 1.5-fold mean batch effect, and a 2-fold dispersion batch effect, we observed a 0.89 TPR from ComBat-seq, which is higher than the other methods (including batch as a covariate: 0.87, original ComBat on logCPM: 0.85, RUV-seq: 0.83, SVA-seq: 0.87). While in a more extreme scenario, the benefit of ComBat-seq is more visible. With a 3-fold mean change and a 4-fold dispersion effect, ComBat-seq achieves a 0.73 TPR, at least 6% higher than the other methods (including batch as a covariate: 0.67, original ComBat on logCPM: 0.66, RUV-seq: 0.61, SVA-seq: 0.66).

Proportions of simulations where each of the five batch correction method achieves the highest power or lowest false positive rates are summarized in Supplementary Figure S3. Supplementary Figure S3 confirms that as the level of batch effect increases, ComBat-seq indeed has a much better chance than the other methods to achieve high detection power, while controlling false positive rates.

### Application to the GFRN signature dataset

**Figure 4.**
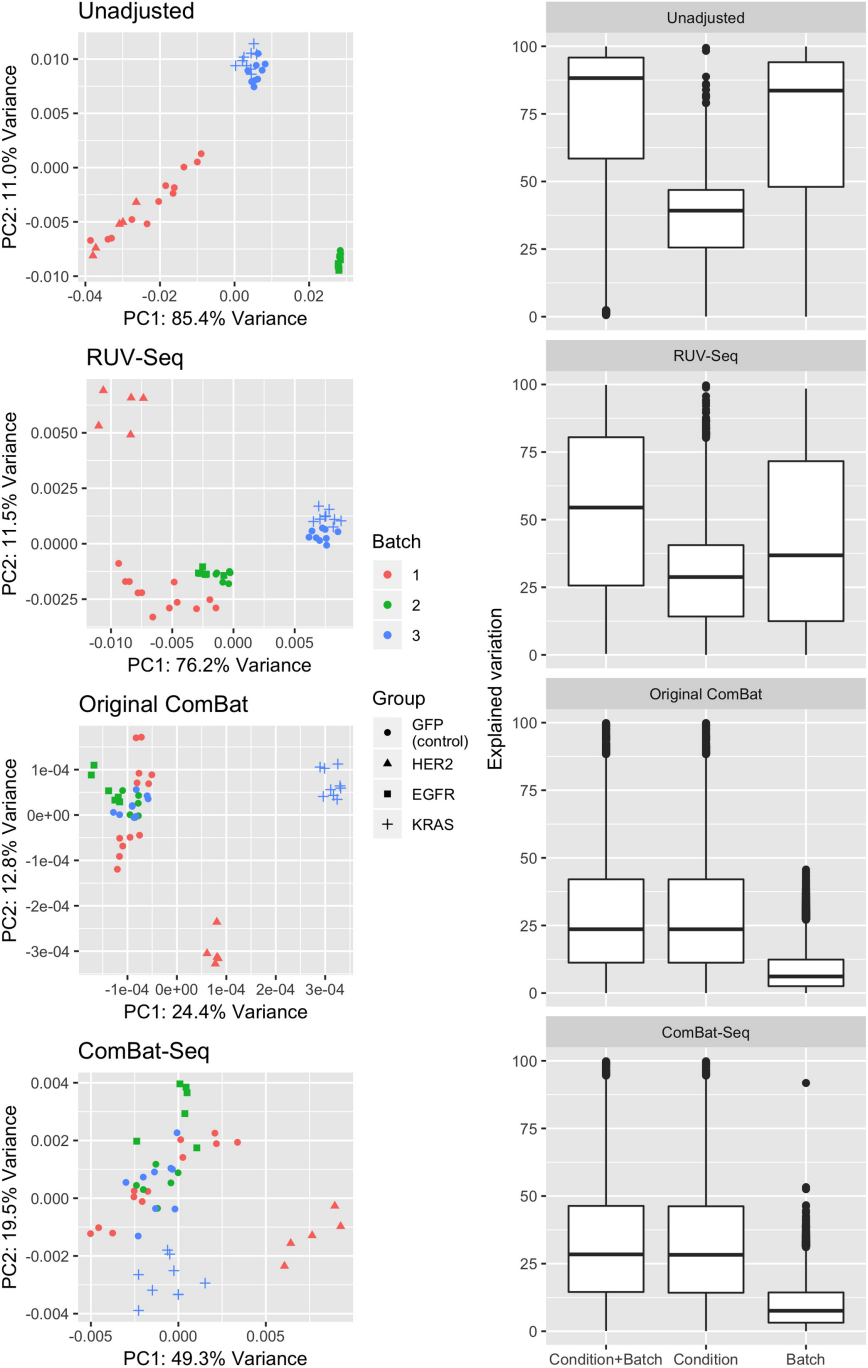
Application of ComBat-seq for removing batch effects in a pathway activation dataset. The unadjusted data contains a strong batch effect, as samples clearly separated by batch in the principal components (top left panel, ‘Unadjusted’). An effective adjustment is expected to bring control samples from the three batches to the same level, while maintaining biological signals from the different treated samples, each of which is only present in a single batch. We observed that in the PCA plots, ComBat-seq is able to recover the expected biological pattern, while RUV-seq was not able to fully address the batch effect. This is further shown in the analysis of explained variation in unadjusted data, and in data adjusted by ComBat-seq and RUV-seq. In the ComBat-seq adjusted data, variation explained by batch is greatly reduced compared to that in unadjusted data. Though ComBat-seq does not show improved results in this example than the current model used on logCPM, we emphasize its benefits in increased statistical power in differential expression than the current ComBat, as we have shown in the simulation studies.

We applied our ComBat-seq approach to address batch effects in a real RNA-seq dataset designed to develop pathway signatures for breast cancer progression and treatment response (13) as described in the ‘Materials and Methods’ section. Figure 4 shows the scatter plot of samples projected on the first two principle components in unadjusted data, and in data adjusted by RUV-seq, ComBat-seq, and using the original ComBat on logCPM. We observed a strong batch effect in the unadjusted data, which was not fully addressed by RUV-seq. For the result in Figure 4, we specified a single latent factor in RUV-seq. We further note that increasing the number of latent factors does lead to better separation between GFP controls and activated samples (*K* = 2, Supplementary Figure S4), but it does not clearly separate GFP controls from all treated samples, as we see for ComBat-seq. In the PCA of ComBat-seq adjusted data, we observed the expected pattern of data if there were no batch effects, in which the control samples are clustered together, while the treated samples from three conditions are scattered at different locations. The effective adjustment of ComBat-seq is further shown in the boxplot of proportion of explained variation by condition and batch across genes. In ComBat-seq adjusted data, variation explained by batch is greatly reduced compared to that in the unadjusted data. These results suggest a successful adjustment of batch effect from ComBat-seq.

Though ComBat-seq does not show clearly improved results compared to using ComBat on logCPM in this application example, we re-emphasize that as shown in the simulations, using ComBat-seq which preserves integer counts instead of log-transforming the data results in better statistical power in differential expression.

We further evaluated DE genes, comparing treated samples to pooled GFP controls from the three batches. The top 100 DE genes for each condition, under each batch adjustment situation, are given in Supplementary Data S1–3. The number of detected DE genes, as well as their comparative statistics are summarized in Supplementary Data S4. In summary, ComBat and ComBat-seq tend to have relatively large overlap in DE genes, which is expected because they use the same underlying linear model specification.

These data were derived using samples from quiescent human mammary epithelial cells that were transfected with adenoviruses expressing either one of HER2, EGFR, KRAS or (in controls) GFP. This means that these specific genes should clearly be overexpressed in these samples. As expected, HER2 and KRAS are constantly ranked at the top do the list in the DE genes for all methods, including the unadjusted data. However, due to the batch effect between GFP controls, EGFR is not detected as DE in the unadjusted data using an FDR ≤ 0.05. In data adjusted by RUV-seq or SVA-seq, EGFR is not detected either, regardless of the number of latent factors/surrogate variables used. In data adjusted by the ‘one-step’ approach, ComBat and ComBat-seq, EGFR is found to be DE using an FDR ≤ 0.05. We also looked at the relative percentile ranking of EGFR in the DE gene list (FDR adjusted *P*-values inclded as well): ‘one-step’: ranked 94.3% (FDR = 0.043); ComBat: ranked 26.6% (FDR = 0.0003); ComBat-seq: ranked 42.3% (FDR = 0.0016). The expression values, in logCPM, of EGFR is included in Supplementary Figure S5. Note that SVA-seq and the ‘one-step approach’ do not generate adjusted data, and therefore, expression of EGFR is not shown for these two methods.

In addition to the genes mentioned above, we also expect to see changes in their downstream targets. For example, for KRAS, we took the genes in RAS signaling pathway (15), and compared their rankings in the differential expression analysis results across different batch adjustment methods. In general, data adjusted for batch effects tend to rank genes in the RAS signaling pathway higher than the unadjusted data. In the top 1000 genes, the one-step approach found 25 genes (Fisher’s exact test for enrichment, *P* = 0.002) and ComBat-seq found 24 (*P* = 0.004) of the 222 genes in RAS signaling pathway. SVA-seq found 21 genes (*P* = 0.029), followed by original ComBat with 20 genes (*P* = 0.043), and RUVSeq with 19 genes (*P* = 0.080). There were only 16 RAS genes found in unadjusted data results (*P* = 0.306). These results suggest that batch correction approaches, including ComBat-seq are able to increase the amount of meaningful biological knowledge than can be obtained from the data.

## DISCUSSION

We developed ComBat-seq to adjust batch effects from known sources in count data from RNA-seq studies. ComBat-seq is able to preserve the integer nature of count data, making the analysis pipeline more compatible for RNA-seq studies. We showed in simulations that ComBat-seq generally out-performs other methods in terms of the impact on downstream differential expression. When variance batch effect is present in the data, ComBat-seq is able to achieve better statistical power, while controlling false positive rates, compared to the other available methods. We further demonstrated the utility of ComBat-seq in addressing batch effect in the GFRN signature dataset, showing its potential to recover biological signals from data affected by batch.

ComBat-seq is an extension of the original ComBat approach. Both methods use similar linear models to describe gene expression and parameterize the expression with background level, changes caused by biological condition and mean and variance batch effect. These parameters are estimated, and used for adjusting batch effect.

The differences in the underlying probabilistic assumption leads to the discrepancies in estimation and adjustment between ComBat-seq and original ComBat. The original ComBat assumes Gaussian distribution, which is more sensitive to outlying data points. Therefore, ComBat uses empirical Bayes shrinkage, pooling information across genes to generate more robust estimates for gene-wise mean and variance batch effect. In ComBat-seq, however, data are assumed to follow a Negative Binomial distribution, which is a more flexible family of models that can better handle outliers and skewed data. Estimates are directly obtained from the negative binomial regression model, using standard Fisher scoring approach. We have shown (in Supplementary Material) that applying empirical Bayes shrinkage is not necessary for ComBat-seq because the approach is already sufficiently robust due to the distributional assumption.

An additional difference between ComBat and ComBat-seq lies in the adjustment approach. ComBat removes batch effect from the data via standardization, i.e. subtracting the mean batch effect estimate, and scaling by variance estimates. This is mathematically equivalent to quantile-matching for Gaussian data. However, adjusting the quantiles of Negative Bionomial data is not as straightforward and cannot be accomplished by directly standardizing the data. Thus, the adjustment method in ComBat-seq resembles quantile normalization, i.e. mapping the empirical distribution of count data to a expected ‘batch-free’ distribution. This method ensures that the adjusted data remain integer counts, and thus are compatible as input for downstream differential expression software like edgeR and DESeq2.

In simulations, we observed that when there is no true difference in dispersion across batch, applying ComBat-seq, which specifies different dispersion parameters for batches, results in increased false positive rates compared to the other methods without further increasing the detection power. ComBat-seq controls false positives and shows benefits in increased true positive rates only when a true dispersion batch effect is present in the data. This is consistent with the intuition of batch effect adjustment, that modifying the data in any way comes with a risk of jeopardizing biological signals in the data. Therefore, batch effects should only be adjusted when they are present and result in unfavorable impact on downstream analysis. Such observations emphasize the importance for careful diagnosis of batch effect before applying any transformation to the data, which our reiterates previous work in this area (16).

In the simulation studies, we also compared ComBat-seq with the commonly applied approach to transform the count matrix to logCPM, and then applied batch correction methods based on Gaussian distributions. That method essentially assumes a log-normal distribution for the data. In simulations, we observed that ComBat-seq generally out-performs log transforming the data in terms of power and control of false positives. These results provide evidence that using appropriate probabilistic models for count data may be more beneficial than arbitrarily transforming the data.

Our study has several limitations. We used an idealistic data model in simulations, and characterized biological signals and batch effects in the form of fold changes in the average value across batch. Though there may be other methods to model count data with both condition and batch effects, our model is a valid and convenient assumption for the data, which is easy to implement with the *polyster* package. We focused primarily on addressing the unwanted impact of batch effect on downstream differential expression. It is known that batch effects may also negatively impact other biological tasks, such as developing predictive models for genomic data. Performance of batch correction in these tasks requires further evaluation, but is beyond the scope of this paper. Our ComBat-seq method is based on a gene-wise negative binomial regression model, which, similar to other (generalized) linear models, may not work well on data with severely or even completely confounded study designs. However, batch correction in confounded designs is challenging for most if not all the state-of-the-art batch adjustment methods, and careful experimental design has been widely advised to mitigate the unfavorable impact of batch effects.

## REPRODUCIBILITY

The ComBat-seq software is available in the sva package in the Bioconductor project (17). Code to reproduce the results in this paper are available at https://github.com/zhangyuqing/ComBat-seq.

## Supplementary Material

lqaa078_Supplemental_Files

## References

[1] Leek J.T., Scharpf R.B., Bravo H.C., Simcha D., Langmead B., Johnson W.E., Geman D., Baggerly K., Irizarry R.A. Tackling the widespread and critical impact of batch effects in high-throughput data. Nat. Rev. Genet. 2010; 11:733–739. 20838408 10.1038/nrg2825 PMC3880143

[2] Robinson M.D., Oshlack A. A scaling normalization method for differential expression analysis of rna-seq data. Genome Biol. 2010; 3:R25. 10.1186/gb-2010-11-3-r25 PMC2864565 20196867

[3] Risso D., Ngai J., Speed T.P., Dudoit S. Normalization of rna-seq data using factor analysis of control genes or samples. Nat. Biotechnol. 2014a; 32:896–902. 25150836 10.1038/nbt.2931 PMC4404308

[4] Johnson W.E., Li C., Rabinovic A. Adjusting batch effects in microarray expression data using empirical bayes methods. Biostatistics. 2007; 8:118–127. 16632515 10.1093/biostatistics/kxj037

[5] Leek J.T. Svaseq: removing batch effects and other unwanted noise from sequencing data. Nucleic Acids Res. 2014; 42:e161. 10.1093/nar/gku864 PMC4245966 25294822

[6] Zhang Y., Jenkins D.F., Manimaran S., Johnson W.E. Alternative empirical bayes models for adjusting for batch effects in genomic studies. BMC Bioinformatics. 2018; 19:262. 30001694 10.1186/s12859-018-2263-6 PMC6044013

[7] Robinson M.D., McCarthy D.J., Smyth G.K. edger: a bioconductor package for differential expression analysis of digital gene expression data. BMC Bioinformatics. 2010; 26:139–140. 10.1093/bioinformatics/btp616 PMC2796818 19910308

[8] Love M.I., Huber W., Anders S. Moderated estimation of fold change and dispersion for rna-seq data with deseq2. Genome Biol. 2014; 15:550. 25516281 10.1186/s13059-014-0550-8 PMC4302049

[9] Law C.W., Chen Y., Shi W., Smyth G.K. voom: precision weights unlock linear model analysis tools for rna-seq read counts. Genome Biol. 2014; 15:R29. 24485249 10.1186/gb-2014-15-2-r29 PMC4053721

[10] McCarthy D.J., Chen Y., Smyth G.K. Differential expression analysis of multifactor rna-seq experiments with respect to biological variation. Nucleic Acids Res. 2012; 40:4288–4297. 22287627 10.1093/nar/gks042 PMC3378882

[11] Chen Y., Lun A.T., Smyth G.K. Datta S., Nettleton D. Differential expression analysis of complex RNA-seq experiments using edger. Statistical Analysis of Next Generation Sequencing Data. 2014; Chem Springer 51–74.

[12] Frazee A.C., Jaffe A.E., Langmead B., Leek J.T. Polyester: simulating rna-seq datasets with differential transcript expression. Bioinformatics. 2015; 31:2778–2784. 25926345 10.1093/bioinformatics/btv272 PMC4635655

[13] Rahman M., MacNeil S.M., Jenkins D.F., Shrestha G., Wyatt S.R., McQuerry J.A., Piccolo S.R., Heiser L.M., Gray J.W., Johnson W.E. et al. Activity of distinct growth factor receptor network components in breast tumors uncovers two biologically relevant subtypes. Genome Med. 2017; 9:40. 28446242 10.1186/s13073-017-0429-x PMC5406893

[14] McQuerry J.A., Jenkins D.F., Yost S.E., Zhang Y., Schmolze D., Johnson W.E., Yuan Y., Bild A.H. Pathway activity profiling of growth factor receptor network and stemness pathways differentiates metaplastic breast cancer histological subtypes. BMC Cancer. 2019; 19:881. 31488082 10.1186/s12885-019-6052-z PMC6727561

[15] RAS Pathway v2.0 National Cancer Institute. 2020; (03 August 2020, date last accessed) https://www.cancer.gov/research/key-initiatives/ras/ras-central/blog/2015/ras-pathway-v2.

[16] Manimaran S., Selby H.M., Okrah K., Ruberman C., Leek J.T., Quackenbush J., Haibe-Kains B., Bravo H.C., Johnson W.E. BatchQC: interactive software for evaluating sample and batch effects in genomic data. Bioinformatics. 2016; 32:3836–3838. 27540268 10.1093/bioinformatics/btw538 PMC5167063

[17] Leek J.T., Johnson W.E., Parker H.S., Jaffe A.E., Storey J.D. The sva package for removing batch effects and other unwanted variation in high-throughput experiments. Bioinformatics. 2012; 28:882–883. 22257669 10.1093/bioinformatics/bts034 PMC3307112

