# Supplementary material for: ComBat-seq: batch effect adjustment for RNA-seq count data
Source: NAR Genom Bioinform. 2020 Sep 21;2(3):lqaa078. doi: 10.1093/nargab/lqaa078 (PMC7518324; doi:10.1093/nargab/lqaa078)
Supplement: lqaa078_Supplemental_Files [file lqaa078_supplemental_files.zip › ComBat_Seq_NAR_supplement_revision080920.pdf]

Supplementary Materials for "*ComBat-Seq*: batch effect adjustment for RNA-Seq count data"

Yuqing Zhang, Giovanni Parmigiani, and W. Evan Johnson

Parameter estimation with shrinkage

Genomic data often have small sample sizes, which makes it challenging to estimate the parameters in the ComBat-Seq model. One of the advantageous features of ComBat is the hierarchical empirical Bayes modeling, which pools information across genes for parameter estimation, making the estimates more robust for data with small sample sizes and/or outlying values Johnson *et al.* (2007). Similar methods for estimation with small sample size have been proposed for negative binomial regression models used on count data. The edgeR software uses a similar idea in its model, which is to estimate the dispersion parameter by maximizing a weighted likelihood, combining both gene-wise likelihood, and likelihood assuming universal dispersion across genes Chen *et al.* (2014).

In ComBat-Seq, we include a similar option to estimate parameters, which is inspired by the non-parametric empirical Bayes method in ComBat. In the ComBat-Seq function, users may select to use this alternative approach by setting the "shrink" parameter to TRUE (default FALSE).

The underlying methods are as follows. After fitting a gene-wise negative binomial regression model, and obtaining the batch effect parameters $(\hat{\gamma}_{gi}, \hat{\phi}_{gi})$, we adjust these parameters as a

weighted average of estimates across genes:

$$\gamma^*_{gi} = \frac{\sum_{k \neq g} \omega_{ki} \hat{\gamma}_{ki}}{\sum_{k \neq g} \omega_{ki}} \tag{1}$$

$$\phi^*_{gi} = \frac{\sum_{k \neq g} \omega_{ki} \hat{\phi}_{ki}}{\sum_{k \neq g} \omega_{ki}} \tag{2}$$

where the weights are defined as

$$\omega_{ki} = L(\bar{Y}_{gi} | \hat{\gamma}_{ki}, \hat{\phi}_{ki}) = \prod_{j=1}^{n_i} d(Y_{gij} | \hat{\gamma}_{ki}, \hat{\phi}_{ki}) \tag{3}$$

d represents the density function for negative binomial distributions. These formulae are directly adopted from those for the posterior estimates of mean and variance batch effect parameters in ComBat. Parameters will be estimated with the gene-wise estimates from all the other genes aside from itself. Estimates with a larger likelihood for the data will be assigned a higher weight. We then calculate the "batch-free" distribution, using the estimates as defined above. Adjusted data are generated same as before.

We evaluated this approach on the pathway signature dataset, using only the control samples from the three batches. Expected adjustment should pool all samples together. We observed that applying shrinkage to the parameter estimates tend to results in under-estimated batch effect, which leads to an under-correction of the data. Batch effects are still present in the data even after adjustment (Figure S1).

We would like to point out, however, that our proposed model is a naive extension from the ComBat non-parametric estimation approach. Deriving an empirical Bayesian approach with negative binomial distributed count data is challenging, for there is no conjugate distributions available. A full Bayesian approach may be feasible, but requires complicated computation and is beyond the scope of our work. We encourage further exploration on the impact of outlying counts in RNA-Seq data, and the benefits of shrinkage in batch effect estimation and adjustment.

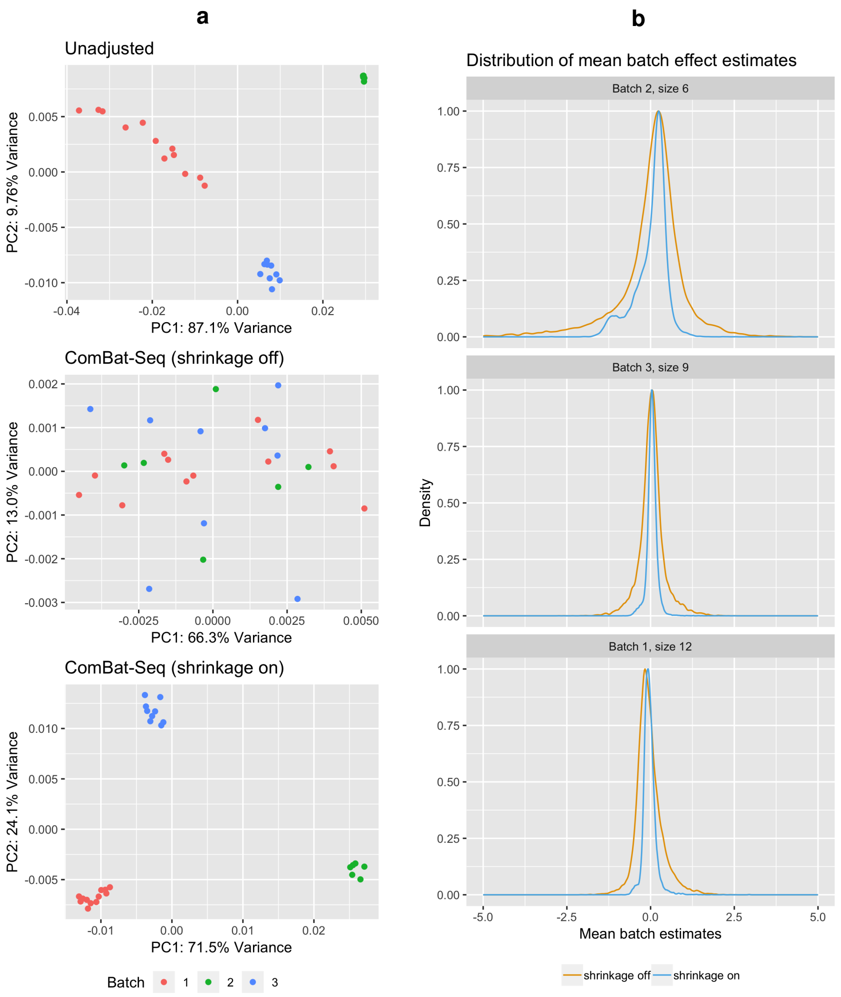

Figure S1: Comparison between estimation with and without shrinkage. We applied the ComBat-Seq model both with and without shrinkage on the GFRN pathway signature dataset, using only the control samples. **a)** When shrinkage is used, the estimated mean batch effect parameters of all genes tend to have a more concentrated distribution, centered at zero. This suggests that the mean batch effects are estimated to be closer to zero, an under-estimation compared to the results using ComBat-Seq without shrinkage. The under-estimation of batch effects leads to **b)** an under-correction, as shown in the PCA plots. Samples still clearly separated by batch after adjustment with the shrinkage version.

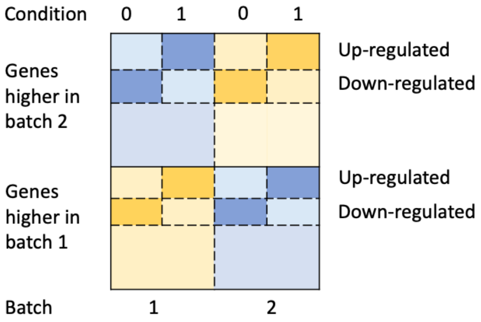

Figure S2: Study design of the simulation experiments. The data are in the form of gene by sample matrices. We simulated 2 biological conditions: negative (0) and positive (1), as well as 2 batches. We also simulated 2 groups of genes. Group 1 has higher expression in batch 2 and lower in batch 1, while group 2 has the reversed pattern, higher in batch 1 and lower in batch 2. The figure shows high expression in yellow, and low expression in blue. We also simulated differentially expressed genes in both groups. A deeper color represents increased expression due to biological condition.

Table S1: Levels of biological and batch effects in real datasets. For the condition effect, we used edgeR to perform differential expression within one of the batches in the studies, and identified the top-50 up-regulated, and top-50 down-regulated genes, ranked by FDR corrected P values. We took the median of fold changes across conditions among the top-50 up-regulated genes, and the median of those among the down-regulated genes. We reported the maximum of two medians in the table. For mean batch effects, we calculated the gene-wise average expression within each batch, and each biological condition. We took the median of mean expression across genes, then compared the medians across batch, and report the maximum fold change. For dispersion differences, we report the maximum fold change in the median gene-wise dispersion across batch. Gene-wise dispersion are estimated with edgeR.

Dataset	Condition	Batch Mean	Batch Dispersion	References
GFRN	1.65	1.88	2.78	Rahman *et al.* (2017)
CHD8	2.92	1.68	7.09	Sugathan *et al.* (2014)
TB	3.98	1.62	1.26	Zak *et al.* (2016); Suliman *et al.* (2018)

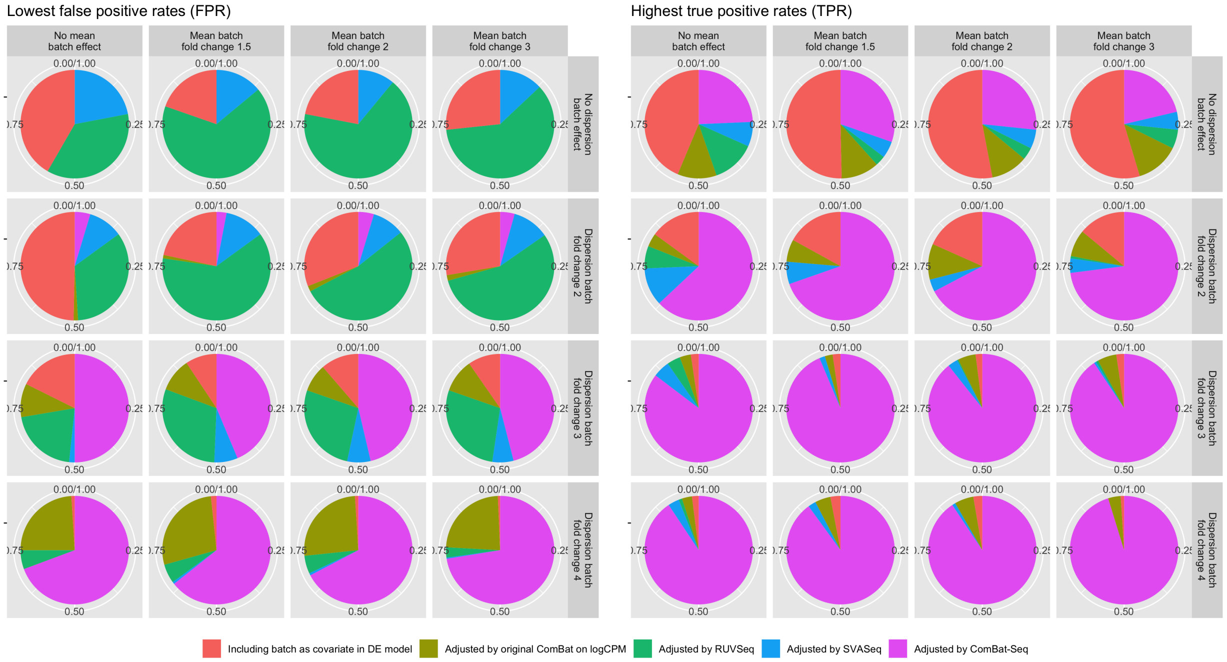

Figure S3: Proportions of simulations where each method achieves the highest true positive rates / lowest false positive rates among the five tested methods. As the level of dispersion difference increases, ComBat-Seq becomes more conservative in controlling false positives, while has better chance to achieve high power for detection than the other methods.

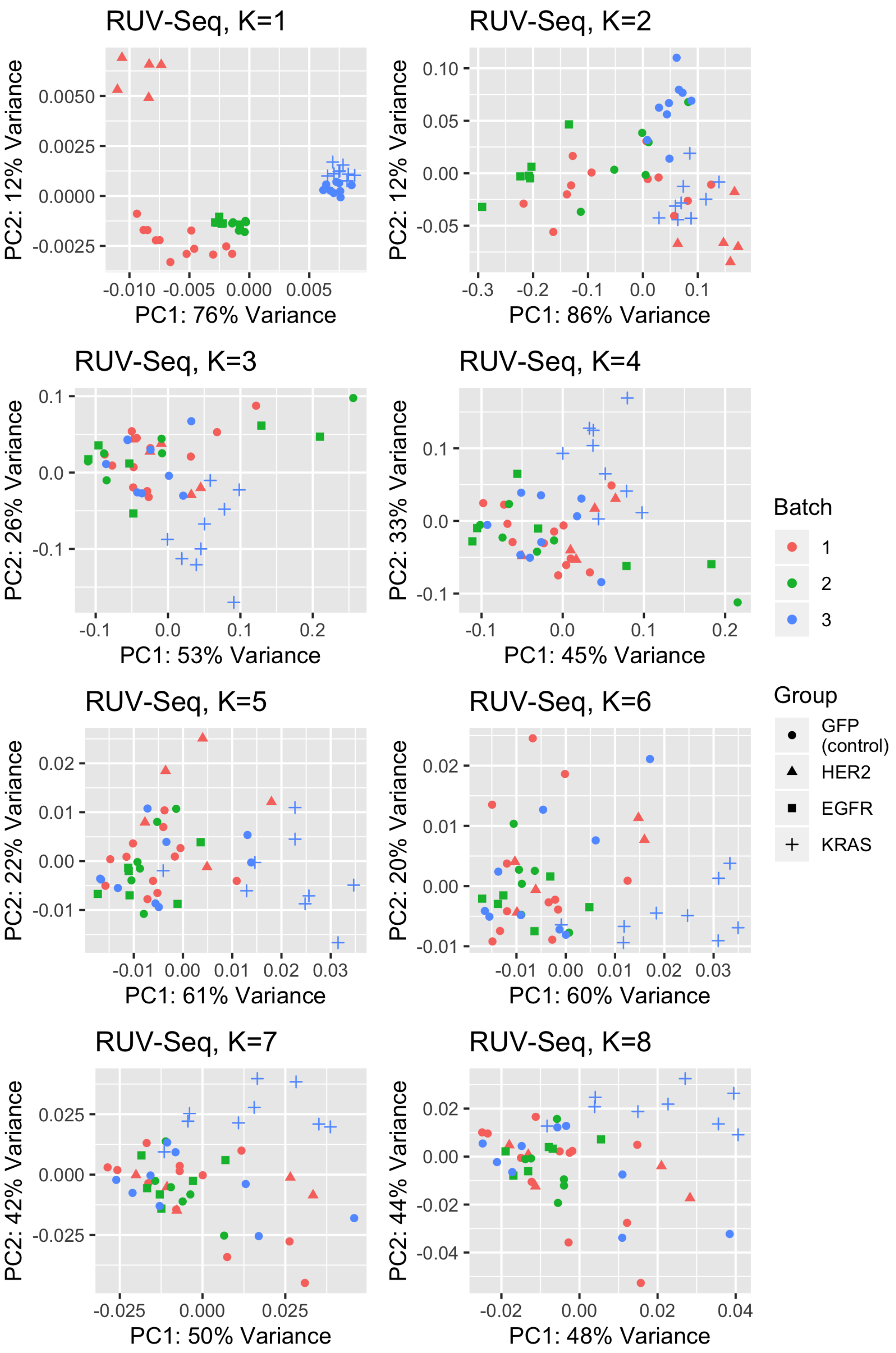

Figure S4: The effect of the number of latent factors on PCA result after RUV-Seq adjustment. In Figure 4 in the main paper, we showed data after RUV-Seq adjustment using a single latent factor (K=1). We explored other choices of number of latent factors. None of these choices achieve a clean separation between GFP controls and the treated samples, as ComBat-Seq achieves.

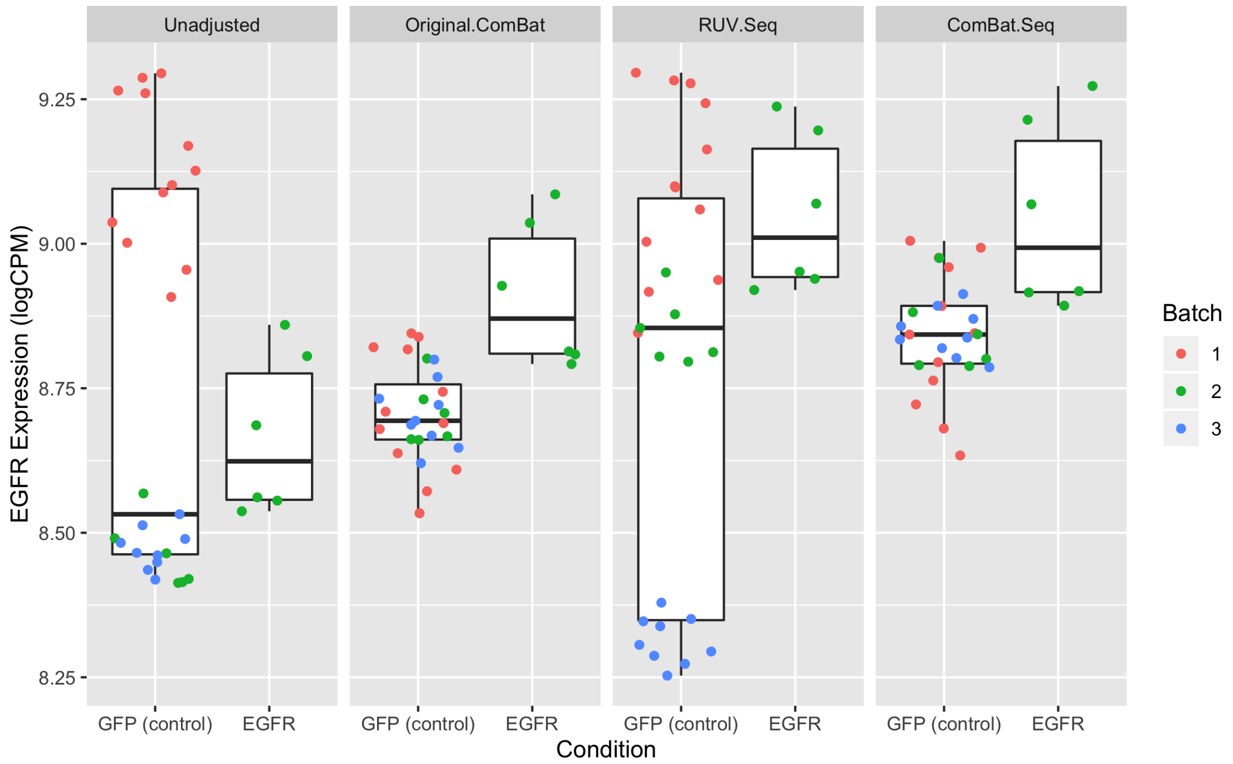

Figure S5: Expression of EGFR in unadjusted data and in data adjusted by original ComBat, RUVSeq, and ComBat-Seq. Results for original ComBat are plotted directly from adjusted data. In the unadjusted data, and data adjusted by RUV-Seq and ComBat-Seq, the skewed count data is transformed into logCPM for better visualization, and comparison to the original ComBat. In unadjusted data, due to the batch effect, the GFP control from batch 1 have higher expression than the treated EGFR samples. As a result, EGFR is not detected as differentially expressed. RUV-Seq was not able to resolve the batch effect satisfactorily. Figure shows using 1 latent factor, while when using 2 factors EGFR remains undetected. ComBat and ComBat-Seq are able to resolve the batch effect in GFP controls, and recover the biological signal for EGFR. It is detected as differentially expressed for both methods.

References

Chen, Y., Lun, A. T., and Smyth, G. K. (2014). Differential expression analysis of complex rna-seq experiments using edger. In *Statistical analysis of next generation sequencing data*, pages 51–74. Springer.

Johnson, W. E., Li, C., and Rabinovic, A. (2007). Adjusting batch effects in microarray expression data using empirical bayes methods. *Biostatistics*, **8**(1), 118–127.

Rahman, M., MacNeil, S. M., Jenkins, D. F., Shrestha, G., Wyatt, S. R., McQuerry, J. A., Piccolo, S. R., Heiser, L. M., Gray, J. W., Johnson, W. E., *et al.* (2017). Activity of distinct growth factor receptor network components in breast tumors uncovers two biologically relevant subtypes. *Genome medicine*, **9**(1), 40.

Sugathan, A., Biagioli, M., Golzio, C., Erdin, S., Blumenthal, I., Manavalan, P., Ragavendran, A., Brand, H., Lucente, D., Miles, J., *et al.* (2014). Chd8 regulates neurodevelopmental pathways associated with autism spectrum disorder in neural progenitors. *Proceedings of the National Academy of Sciences*, **111**(42), E4468–E4477.

Suliman, S., Thompson, E. G., Sutherland, J., Weiner 3rd, J., Ota, M. O., Shankar, S., Penn-Nicholson, A., Thiel, B., Erasmus, M., Maertzdorf, J., *et al.* (2018). Four-gene pan-african blood signature predicts progression to tuberculosis. *American journal of respiratory and critical care medicine*, **197**(9), 1198–1208.

Zak, D. E., Penn-Nicholson, A., Scriba, T. J., Thompson, E., Suliman, S., Amon, L. M., Mahomed, H., Erasmus, M., Whatney, W., Hussey, G. D., *et al.* (2016). A blood rna signature for tuberculosis disease risk: a prospective cohort study. *The Lancet*, **387**(10035), 2312–2322.
